# Apolipoprotein E Gene Polymorphism and Serum Lipid Profile in Saudi Patients with Psoriasis

**DOI:** 10.1155/2014/239645

**Published:** 2014-03-23

**Authors:** Fahad Al Harthi, Ghaleb Bin Huraib, Abdulrahman Zouman, Misbahul Arfin, Mohammad Tariq, Abdulrahaman Al-Asmari

**Affiliations:** ^1^Department of Dermatology, Prince Sultan Military Medical City, P.O. Box 7897, Riyadh 11159, Saudi Arabia; ^2^Research Center, Prince Sultan Military Medical City, P.O. Box 7897, Riyadh 11159, Saudi Arabia

## Abstract

*Background/Aim*. Apolipoprotein E (APOE) gene variants have been reported to influence psoriasis risk. However, data is limited to a few ethnicities and no similar study has been performed in middle eastern populations. We investigated this association in Saudi psoriasis patients. *Methods*. Saudi subjects (294) were genotyped for APOE gene using APOE StripAssay kit. *Results*. The frequencies of alleles **ε**2, **ε**4, and genotypes **ε**3/**ε**4 and **ε**3/**ε**2 were significantly higher in psoriasis patients compared with those in controls. The frequency of **ε**3 allele and **ε**3/**ε**3 genotype was significantly lower in patients. Other genotypes, **ε**2/**ε**4, **ε**2/**ε**2, and **ε**4/**ε**4, were absent in both groups. The serum cholesterol, triglycerides, and LDL levels were significantly higher in psoriasis patients contrary to HDL level. Patients with APOE **ε**4 had significantly higher levels of total cholesterol and LDL cholesterol, whereas those with the **ε**2 had higher HDL cholesterol, and triglycerides. *Conclusion*. APOE alleles **ε**2, **ε**4, and genotypes **ε**2/**ε**3 and **ε**4/**ε**3 are associated with psoriasis and can be a risk factor while allele **ε**3 and genotype **ε**3/**ε**3 may be protective for psoriasis in Saudis. Results of lipid profile support that psoriasis is one of the independent risk factors for hyperlipidemia and emphasize the need of screening cardiovascular diseases in psoriatic patients.

## 1. Introduction 

Psoriasis is a complex autoimmune disease characterized by chronic recurring reddish patches covered with silvery-white scales. It is a common disease affecting approximately 120–180 million people worldwide [1]. Around 150,000 new cases of psoriasis are reported annually. However, the prevalence of psoriasis varies significantly depending mainly on race, geographic location, genetics, and environmental factors [2–5]. Ethnic factors influence the prevalence of psoriasis greatly; it ranges from 0% in the Samoan population to 12% in Arctic Kasach'ye [6] including 5–10% in northernmost regions of the Soviet Union and Norway, 2-3% in the UK, USA, and Holland, 0.4–2% in the Asian countries [7, 8], and 0–0.3% among North American Indians, Latin American Indians, Mongoloids, and Western Africans [9, 10].

Psoriasis has wide clinical spectra that range from epidermal (scaly) to vascular (thickened) erythematous involvement associated with epidermal hyperproliferation, abnormal keratinocyte differentiation, angiogenesis with blood vessel dilation, and the presence of inflammatory cells in the superficial dermis and epidermis. Increased polymorphonuclear leukocyte levels damage surrounding tissue by releasing reactive oxygen species produced via nicotinamide adenine dinucleotide phosphate (NADPH) oxidase/myeloperoxidase and proteolytic enzymes.

The available data suggest that genetic, metabolic, and immunologic factors play important role in the pathogenesis of psoriasis. The concordance of psoriasis in monozygotic twins is 65–72% versus 15–30% in dizygotic twins [2, 11, 12]. Epidemiological studies have shown that the recurrence of psoriasis in first-degree relatives of affected subjects is about ten times greater than that in general population clearly suggesting the genetic basis of psoriasis. Inheritance being multifactorial, genetic variants in multiple genes interact both with each other and with the environment [13–20].

The abnormal lipid metabolism is considered to be an important hallmark in the etiopathogenesis of psoriasis [21–23]. It has been suggested that continuous separation of psoriatic scales caused the permanent loss of lipids which adversely affects lipid homeostasis. Healthy skin secretes 85 mg of cholesterol within 24 hours, whereas a psoriatic patient loses 1-2 grams of cholesterol with scales during that time [24]. Alterations in plasma lipid and lipoprotein composition including a tendency toward an increase in total cholesterol (TC) and triglyceride (TG) and decrease in high-density lipoprotein cholesterol (HDL-C) levels suggest that psoriasis may associate with the disorders of lipid metabolism [25, 26]. 

Lipid metabolism in the epidermis may be regulated by the expression of apolipoprotein E (APOE), a glycoprotein which is synthesized in a wide variety of extrahepatic tissues including skin [27–29]. APOE demonstrates extraordinary functional diversity and it is essential for the normal transformation and metabolic processes involved in lipoprotein [30, 31]. It plays important roles in remnant lipoprotein clearance, the immune response, cell proliferation, and lymphocyte activation [32, 33]. APOE is implicated in psoriasis by providing protection against some infections, as well as by modulating mitogen-activated T lymphocyte proliferation [33–35]. Cytokines are crucial in human inflammatory and autoimmune disorders and APOE could affect these disorders through interacting with cytokines as well. 

APOE gene is located on chromosome 19 and has three polymorphic variants in human designated as *ε*2, *ε*3, and *ε*4. These variants differ from one another by the presence of either C or T nucleotide at codons 112 and 158. These three alleles encode different APOE isoforms which vary significantly in structure and function including receptor binding capacity and lipid metabolism [36]. As each individual human being carries two allelic copies in a gene, six possible genotypes (*ε*2/*ε*2, *ε*3/*ε*3, *ε*2/*ε*3, *ε*3/*ε*4, *ε*2/*ε*4, and *ε*4/*ε*4) are formed by different combinations of these three alleles [37, 38]. In psoriatic lesions and normal skin, the localization of APOE has been revealed by immunohistochemical methods [39]. The role of the APOE gene in psoriasis is also evident from the fact that in psoriatic skin there is the downregulation of APOE expression and the normalization of APOE levels precedes clinical improvement [40]. Several studies have suggested an association between APOE alleles and genotypes with onset and severity of psoriasis. However, available reports are limited to a few ethnicities and results are contradictory [40–48]. Therefore, in the present study, an attempt was made to investigate association of alleles and genotypes of APOE gene with psoriasis in Saudi patients.

## 2. Materials and Methods

### 2.1. Subjects

A total of 294 subjects including 94 psoriasis patients and 200 age and sex matched healthy controls visiting Dermatology Clinic of Prince Sultan Military Medical City, Riyadh, Saudi Arabia, were recruited in this study. All the subjects were biologically unrelated Saudis. The diagnosis of psoriasis was based on skin changes and the location of the condition on the body. All cases and controls were examined and diagnosed by dermatologists. We used a questionnaire to collect demographic and other information. In addition, patients were asked whether they had hand and foot psoriasis and the number of nails affected. On a sketch they indicated how much of their body was affected when their psoriasis was at its worst during the past 2 years. The body was divided into 3 parts, the upper extremities, head and trunk, and lower extremities. Scoring ranged from 0 (0% of the area in one of the regions affected by psoriasis) to 6 (90–100% of the area in one of the regions affected). The head and trunk together and the lower extremities each represented 40% of the body area. Upper extremities represented 20% of the body area. The scoring for the latter was multiplied by 0.5 and the total score index therefore ranged from 0 to 15. Psoriasis was diagnosed based on clinical findings, and the Psoriasis Area and Severity Index (PASI) was determined. The disease was considered as severe in patients with a PASI score = 10. Patients were considered to have “early onset” psoriasis if the onset of the disease was at any age ≤40 years and “late onset” psoriasis if the onset was >40 years. Patients were considered to have “familial” psoriasis if they had at least one first- or second-degree relative affected by the condition. Diagnosis is based on the typical erythematous, scaly skin lesions, often with additional manifestations in the nails and joints. Written informed consent was obtained from all subjects before their enrolment. Among the confirmed 94 cases of psoriasis there were 34 females and 60 males with mean age of 37 ± 15.5 years and the duration of disease ranged from 1 to 20 years with mean duration of 9 ± 4.5 years. Age of onset of disease ranged from 8 to 55 years. The female to male ratio of psoriasis patients in our study was 1 : 1.76. The control group consisted of 50 females and 150 males with mean age of 36 ± 10 years. All the subjects in control group were screened using a questionnaire about the health status and excluded if they had any history of autoimmune disorders. None of the control subjects had a first- or second-degree relative with psoriasis or any autoimmune disorders. This study was approved by the ethical committee of the hospital.

All the patients had clinical and histopathological diagnosis for chronic plaque type psoriasis. The assessment of the severity and extent of disease was done by PASI score [49]. All patients must be diagnosed with plaque psoriasis for at least one year. Exclusion criteria were coexisting inflammatory skin disease, smoking, diabetes mellitus, systemic lupus erythematosus, rheumatoid arthritis, obesity, history of hyperlipidemia, renal and liver failure, hypothyroidism, and systemic therapy. Serum was collected from subjects following 12 hrs of fasting for determination of lipid profile. Lipid profile was determined in controls and patients following a standard automated procedure in Central Pathological Laboratory of Prince Sultan Military Medical City.

### 2.2. Genotyping

Blood was collected in the tubes containing ethylenediaminetetraacetic acid (EDTA) from the psoriasis patients and controls. Genomic DNA was extracted from the blood using QIAamp DNA minikit (Qiagen CA, USA). The genotypes of the APOE polymorphisms were determined using APOE StripAssay kit based on polymerase chain reaction (PCR) and reverse-hybridization technique (ViennaLab Labordiagnostika GmbH, Vienna, Austria). The procedure included three steps: (1) DNA isolation, (2) PCR amplification using biotinylated primers, and (3) hybridization of amplification product to a test strip containing allele-specific oligonucleotide probes immobilized as an array of parallel lines. Bound biotinylated sequences were detected using streptavidin-alkaline phosphatase and color substrates. To cross-check the results the genotypes of the APOE polymorphisms were also determined by PCR and restriction fragment length polymorphism (RFLP) technique. PCR was performed using PuRe Taq Ready-To-Go PCR Beads (GE Healthcare, UK) with the following primers:* forward primer*: 5-GAC GCG GGC ACG GCT GTC CAA GGA GCT GCA GGC GAC GCA GGC CCG GCT GGA CGC GGA CAT GGA GGA-3 and* reverse primer*: 5-AGG CCA CGC TCG ACG CCC TCG CGG GCC CCG GCC TGG TAC ACT-3.

The 200–300 ng of genomic DNA was used as a template in 25 *μ*L reaction. Genomic DNA was amplified for 40 cycles. Each cycle consisted of 94°C for 30 sec, 68°C for 10 sec, and 72°C for 1 min; PCR products obtained were separated by electrophoresis on 1.5% agarose gel in TAE buffer, visualized by ethidium bromide fluorescence. Fragments with the expected size were cut from the gel, purified using GFX PCR DNA Gel band purification kit (GE Healthcare, UK). Purified DNA was digested with* Cfo *I (Hha I) enzyme, separated by agarose gel electrophoresis to identify the genotype. On the basis of size and number of various fragments generated, APOE genotypes were determined as *ε*2/*ε*2 with 144 bp and 96 bp, *ε*3/*ε*3 with 144 bp and 48 bp, *ε*4/*ε*4 with 72 bp and 48 bp, *ε*2/*ε*3 with 144 bp, 96 bp and 48 bp, *ε*3/*ε*4 with 144 bp, 72 bp, and 48 bp, and *ε*2/*ε*4 with 144 bp, 96 bp, 72 bp, and 48 bp fragments. The prevalence of various genotypes in patients and controls was determined. Complete matching of results was obtained following both of the abovementioned procedures.

### 2.3. Statistical Analysis

The lipid profile results were expressed as mean ± standard deviation. A *P* < 0.05 was considered statistically significant. Statistical analysis was performed using the statistical package for social sciences (SPSS-12, Chicago, USA). To evaluate the differences between groups, Student's *t*-test was used.

The differences in genotype and allele frequencies between patients and controls were analyzed with Fisher's exact test using CalcFisher software (http://www.jstatsoft.org/v08/i21/paper).

*P* values of ≤0.05 were considered significant. The strength of the association of disease with respect to a particular genotype/allele was expressed with odds ratio interpreted as relative risk (RR) following the method of Woolf as described by Schallreuter et al. [50]. RR indicates how many times more frequent a disease is in the positive subjects compared with allele/genotype-negative subjects. It is calculated for a genotype/allele that is increased or decreased in psoriasis patients compared to the frequency in normal Saudi subjects. RR was calculated for all the subjects using the following formula:

(1) $$\begin{array}{c} \text{RR}=\frac{\left(a\right)\times \left(d\right)}{\left(b\right)\times \left(c\right)}; \end{array}$$

(*a*) is number of patients expressing the allele or genotype;

(*b*) is number of patients without allele or genotype expression;

(*c*) is number of controls expressing the allele or genotype;

(*d*) is number of controls without allele or genotype expression.

The etiologic fraction (EF) indicates the hypothetical genetic component of the disease. EF values of >0.00–0.99 are significant. It is calculated for positive associations (RR > 1) using the following formula proposed by Svejgaard et al. [51]:

(2) $$\begin{array}{c} \text{EF}=\frac{\left(\text{RR}-1\right)f}{\text{RR}},\;\text{where}\;f=\frac{a}{a+b}. \end{array}$$

Preventive fraction (PF) indicates the hypothetical protective effect of one allele/genotype for a disease. It is calculated for negative associations (RR < 1) using the following formula [51]. Values of <1.0 indicate the protective effect of an allele/genotype against the manifestation of disease:

(3) $$\begin{array}{c} \text{PF}=\frac{\left(1-\text{RR}\right)f}{\text{RR}\;\left(1-f\right)}+f,\;\text{where}\;f=\frac{a}{a+b}. \end{array}$$

## 3. Results

The results of frequency of APOE alleles and genotypes in the psoriasis patients and the control subjects are summarized in Tables 1–3. The frequency of the *ε*3 alleles was significantly lower in the psoriasis patients (85.11%) compared to the control subjects (95.7%, *P* = 0.0001, RR = 0.254, and PF = 0.459). On the other hand the frequency of the *ε*4 allele was significantly higher in the psoriasis patients compared with that in controls (10.64% versus 4.2%, *P* = 0.005, RR = 2.682, and EF = 0.338). The frequency of allele *ε*2 was 4.25% in patients while completely absent in control groups (*P* = 0.0001).

In both, psoriasis patient and control groups the genotype distributions were in Hardy-Weinberg equilibrium. Our study on various genotypes of APOE also showed variations in patient and control groups (Table 2). The frequencies of genotype *ε*3/*ε*4 and *ε*3/*ε*2 were significantly higher in patients (21.28 and 8.51%) compared with those in controls (8.5 and 0%, resp.). Though the *ε*3/*ε*3 genotype was more common in both the test and control Saudi population, the statistical analysis of data showed significant difference in *ε*3/*ε*3 genotype frequencies between patients and controls. Significantly lower frequency of *ε*3/*ε*3 genotype was found in psoriasis patients compared with that in control (*P* = 0.0001, RR = 0.219, and PF = 0.486). Other genotypes, *ε*2/*ε*4, *ε*2/*ε*2, and *ε*4/*ε*4, were absent in both groups. These results indicated that alleles *ε*2 and *ε*4 are associated with psoriasis and can be a risk factor while allele *ε*3 may be protective in Saudis for psoriasis. Allele *ε*3 containing homozygous genotype (*ε*3/*ε*3) is protective, whereas heterozygous genotypes *ε*2/*ε*3 and *ε*4/*ε*3 are susceptible to the psoriasis in Saudis. The frequencies of various genotypes and alleles were almost similar in male and female patients clearly indicating that gender plays no role in APOE genotype/allele distributions in our population (Table 3).

The values of the lipid profile for the control and psoriasis patient are presented in Table 4. The serum total cholesterol, triglycerides, LDL-cholesterol, and TC/HDL ratio were found to be significantly higher in psoriasis patient than in control group. By contrast HDL-cholesterol level was significantly lower in patient group compared to that in controls. Values for BMI and sugar level also varied in two groups.

The lipid profile of psoriasis patients with respect to APOE allele is presented in Table 5. Significant differences were found in the levels of total cholesterol, HDL-cholesterol, LDL-cholesterol, or triglycerides between the patients according to the APOE allele. Patients in the* APOE  ε3* group had 5.71, 1.31, 3.89, and 1.30 (mmol/L) of total cholesterol, HDL cholesterol, LDL cholesterol, and triglycerides, respectively. Comparatively, subjects in the* APOE* *ε4 *group had significantly higher levels of total cholesterol (5.95 mmol/L) and LDL cholesterol (4.12 mmol/L), whereas those in the* APOE* *ε2* group had higher HDL cholesterol (1.49 mmol/L), triglycerides (1.49 mmol/L), and lower total cholesterol (5.32 mmol/L) and LDL cholesterol (3.25 mmol/L) concentrations.

## 4. Discussion 

Our results showed higher frequency of APOE *ε*2 allele and predominance of *ε*2/*ε*3 genotype in the psoriasis patients in comparison with matched controls suggesting that allele *ε*2 carriers are at a higher risk of developing psoriasis. These results are in agreement with the earlier reports on Japanese [41], Chinese [42–44], and Greek populations [40]. Contrary to this another report from Japan indicated no difference in allele frequencies in psoriasis patients and controls [52]. Recently a meta-analysis on association of APOE polymorphism with psoriasis indicated that APOE *ε*2 is associated with increased susceptibility to psoriasis and allele *ε*3 may decrease psoriasis risk [48].

APOE is involved in transport and metabolism of cholesterol, triglyceride, and other lipids. The lipid transporting and catabolic activity in APOE-*ε*2 carriers is significantly slower as compared to *ε*3 and *ε*4 carriers due to lowest receptor binding affinity of *ε*2. Individuals with APOE *ε*2 are unable to efficiently clear lipids from plasma/tissues which facilitates the accumulation of chylomicron, very low density lipoprotein, and lipids [39]. The localization of APOE in plaques has also been confirmed by immunohistochemical methods [39]. Furumoto et al. [41] suggested that APOE protein might be involved in pathogenesis of psoriasis via the sequestration of lipids contributing to the epidermal barrier function. Several investigators have implicated the *ε*2 protein in the synthesis of structural elements of epidermal cornified layer in psoriatic patients [34, 53].

The APOE *ε*2 isoform differs from the APOE *ε*3 isoform by one amino acid, at position 158, with *ε*2 containing cysteine and *ε*3 containing arginine. This single amino acid difference results in markedly reduced binding of APOE *ε*2 to the low density lipoprotein family of receptors, which in turn results in profound metabolic consequences, particularly Type III hyperlipidemia. Additionally the two cysteines in APOE *ε*2 (positions 112 and 158) permit APOE *ε*2 to form disulfide-linked multimeric protein complexes. These unique properties of APOE *ε*2 may contribute to its role in the etiology of lipid associated diseases including psoriasis.

A significantly lower frequency of *ε*3 allele was observed in Saudi psoriasis patients compared with matched controls (*P* = 0.0001) clearly indicating that APOE *ε*3 exerts protection against psoriasis. These results are in accordance with earlier reports from China, Japan, and Greece suggesting that allele *ε*3 decreases the risk of psoriasis [40–44, 54].

Our results also showed a significantly higher frequency of genotype *ε*2/*ε*3 in Saudi psoriasis patients as compared to matched controls (*P* = 0.0001). The genotype *ε*2/*ε*3 has been associated with significant imbalance in lipids and lipoprotein metabolism. APOE *ε*2/*ε*3 genotype has also been associated with ischemic cerebrovascular diseases [55, 56].

Our results also showed higher prevalence of *ε*4 allele in patient group compared to that in controls suggesting that *ε*4 allele may increase the risk of psoriasis. Similarly higher frequency of *ε*4 alleles has been reported in British and Spanish psoriasis patients [45, 47]. These authors suggested that APOE-*ε*4 may influence disease severity in European patients with psoriasis.

The APOE *ε*4 allele is seen as a “thrifty” gene and its important functions include the increase of cholesterol production in the liver and insulin production in the pancreas. Therefore, ApoE4 tends to reduce the level of high-density lipoprotein (HDL) and increase the level of low-density lipoprotein (LDL) in the high-fat intake population [57] which are critical risk factors for occlusive lipid disorders. The implication of APOE *ε*4 in lipid metabolism and developing of immunologic responses to lipid antigens may contribute to psoriasis as reported earlier [34, 35, 58].

The lipid disturbances have been associated with the pathogenesis of psoriasis [5]. In patients with psoriasis, increased frequency of hyperlipidemia has been reported earlier [59, 60]. A number of studies reported increased total cholesterol, LDL cholesterol and/or triglycerides, and decreased HDL cholesterol in psoriatic patients' serum [61, 62]. The present study showed elevated serum total cholesterol, LDL-cholesterol, and triglyceride and lower serum HDL cholesterol levels in psoriasis patients (Table 4), and our findings might reflect the high incidence of atherosclerosis in patients with psoriasis as reported earlier also for the Japanese [41]. High LDL and/or low HDL levels, risk factors for atherosclerosis, are also a common clinical feature in psoriasis. Earlier studies have suggested that patients with psoriasis have an increased risk of various noncutaneous diseases, including arterial and venous occlusive diseases [63, 64].

Our results showed significant differences in the levels of total cholesterol, HDL-cholesterol, LDL-cholesterol, or triglycerides between the patients having different APOE allele. Significantly higher levels of total cholesterol and LDL cholesterol were noticed in the* APOE* *ε4 *group, whereas* APOE  ε2 *group had higher HDL cholesterol and triglycerides concentrations. These results are also in agreement with earlier published reports [65–68]. Genotype *ε*2/*ε*3 has also been associated with type III hyperlipoproteinemia [55].

Our results showed a trend for higher total cholesterol and LDL-cholesterol in *ε*4 carriers than in *ε*3-homozygotes carrier psoriasis patients. Earlier, *ε*4 allele has been associated with higher level of total cholesterol and LDL [65–68]. The APOE *ε*4 has been also linked to lower C-reactive protein (CRP); it has been suggested that the effect on CRP is a consequence of intrinsic functional differences among the *ε*2, *ε*3, and *ε*4 APOE proteins in the plasma [69]. On the basis of this immunomodulatory effect the APOE-*ε*4 has been attributed to psoriasis severity [47].

## 5. Conclusion 

In conclusion, our study showed that APOE polymorphisms are associated with psoriasis and allele *ε*2 is associated with increased susceptibility for psoriasis, whereas allele *ε*3 may be protective for psoriasis in Saudis. In addition, allele *ε*4 may be a risk factor of psoriasis severity. But this relationship between APOE polymorphisms and the risk of psoriasis warrants further confirmation with large-size sample studies. Similar studies on different ethnic populations will be helpful in defining the role of APOE as a putative pharmacological target for psoriasis. Further the elevated serum levels of cholesterol, triglycerides, and LDL in psoriasis support that the psoriasis is one of the independent risk factors for hyperlipidemia and emphasize the need of screening for cardiovascular diseases in Saudi psoriatic patients.

## Figures and Tables

**Table 1:** Apolipoprotein E alleles frequencies in psoriasis patients and matched controls.

Allele	Psoriasis (*N* = 188)		Control (*N* = 400)		*P* value	RR	EF*/PF
	*N*	Freq. %	*N*	Freq. %			
*ε*3	160	85.11	383	95.75	0.0001^†^	0.254	0.459
*ε*4	20	10.64	17	4.25	0.0050^†^	2.682	0.338*
*ε*2	8	4.25	0	0	0.0001^†^	—	—

^†^Statistically significant; *N*: number of alleles; RR: relative risk; EF: etiological fraction; PF: preventive fraction.

**Table 2:** Apolipoprotein E genotypes frequencies in psoriasis patients and matched controls.

Genotype	Psoriasis (*N* = 94)		Control (*N* = 200)		*P* value	RR	EF*/PF
	*N*	Freq. %	*N*	Freq. %			
*ε*3/*ε*3	66	70.21	183	91.50	0.0001^†^	0.219	0.486
*ε*3/*ε*4	20	21.28	17	8.50	0.0040^†^	2.910	0.354*
*ε*3/*ε*2	8	8.51	0	0	0.0001^†^	—	—
*ε*2/*ε*4	0	0	0	0	—	—	—
*ε*2/*ε*2	0	0	0	0	—	—	—
*ε*4/*ε*4	0	0	0	0	—	—	—

^†^Statistically significant; *N*: number of subjects; RR: relative risk; EF: etiological fraction; PF: preventive fraction.

**Table 3:** Apolipoprotein E allele/genotype frequencies in male/female psoriasis patients and controls.

Genotype/alleles	Male patients (*N* = 60)	Female patients (*N* = 34)	Controls (*N* = 200)
	Freq. (%)	Freq. (%)	Freq. (%)
*ε*3/*ε*3	75.00*	61.76*	91.50
*ε*3/*ε*4	20.00*	23.53*	8.50
*ε*3/*ε*2	5.00*	14.71*	0
*ε*2/*ε*4	0	0	0
*ε*2/*ε*2	0	0	0
*ε*4/*ε*4	0	0	0
*ε*3	87.50*	80.88*	95.75
*ε*4	10.00*	11.77*	4.25
*ε*2	2.50*	7.35*	0

*P value < 0.05 as compared to the frequency in controls; *N*: number of subjects.

*P* values are not significant (>0.05) when frequencies in male were compared with those in female patients.

**Table 4:** Lipid profile of patients and controls.

Parameters	Psoriasis	Controls
Male : female	60 : 34	60 : 40
Mean age (years)	37 ± 15.5	36 ± 10
Total cholesterol (mmol/L)	5.569 ± 0.997*	4.973 ± 0.909
Triglyceride (mmol/L)	1.401 ± 0.776*	1.146 ± 0.554
LDL cholesterol(mmol/L)	3.455 ± 0.906*	2.748 ± 0.569
HDL cholesterol (mmol/L)	1.383 ± 0.310	1.528 ± 0.218*
TC/HC ratio	4.39 ± 2.1	3.3 ± 1.5
Blood sugar (mmol/L)	6.105 ± 2.425	5.551 ± 0.665
BMI	24.0 ± 5.50	22.5 ± 2.21

*P < 0.05.

**Table 5:** Lipid profile in psoriasis patients with APOE allele groups.

Carriers of APOE allele	Total cholesterol (mean ± SD)	HDL cholesterol (mean ± SD)	LDL cholesterol (mean ± SD)	Triglycerides (mean ± SD)
*ε*2	5.36 ± 0.08	1.49 ± 0.03*	3.25 ± 0.07	1.49 ± 0.05*
*ε*3	5.71 ± 0.05	1.31 ± 0.02	3.89 ± 0.03	1.30 ± 0.04
*ε*4	5.95 ± 0.07*	1.27 ± 0.02	4.12 ± 0.08*	1.35 ± 0.06

All values are in mmol/L; *higher than other groups.
